# Exploring Caregiver Perceptions of Child Sleep Quality Among a Racially and Ethnically Diverse Sample: A Qualitative Thematic Analysis

**DOI:** 10.3390/children13050662

**Published:** 2026-05-09

**Authors:** Abby P. M. Katz, Madelyn Dewitt, Naomi Zeltzer, Bethel Daniel, Brooke Ury, Zoe Maxwell, Aliana Rodriguez Acevedo, Huy Tran, Isha Thakkar, Diana S. Grigsby-Toussaint

**Affiliations:** 1 Department of Behavioral and Social Sciences, Brown University School of Public Health, Providence, RI 02912, USA; abby_katz@brown.edu (A.P.M.K.);; 2 Center for Health Promotion and Health Equity, Brown University School of Public Health, Providence, RI 02912, USA; 3 Warren Alpert Medical School, Brown University, Providence, RI 02912, USA; 4 Department of Epidemiology, Brown University School of Public Health, Providence, RI 02912, USA; 5 Institute at Brown for Environment and Society, Brown University, Providence, RI 02912, USA

**Keywords:** sleep quality, sleep hygiene, sleep disparities, children, parenting, qualitative analysis, thematic analysis, template analysis

## Abstract

**Background:** High quality pediatric sleep is shaped by multiple factors, including duration, restoration, and continuity. Multiple socio-ecological factors that are typically enforced by caregivers (e.g., bedtime routines) also determine the likelihood of attaining optimal pediatric sleep health. Consistent with the extant sleep literature on pre-pubertal children, this qualitative study targeted caregivers to identify factors influencing children’s sleep quality. **Methods:** Participants were recruited from Project G-SPACE, a US-based study exploring the influence of greenspace on sleep and mental health among elementary school-aged children. A racial, ethnic, and socio-economically diverse sample of caregivers (*n* = 21) participated in virtual semi-structured interviews about their perceptions of determinants of child sleep quality and behavior. Template-style thematic analysis was employed to synthesize the interviews. **Results:** Caregivers report that busy days for their children, especially characterized by high levels of physical activity, facilitate sleep continuity and good sleep quality. Sibling dynamics can be disruptive, resulting in poor sleep quality. To promote sleep health, parents employ rules regarding screentime, food/drink, and bed/wake time schedules, though the latter seems to be more flexible when children are not in school (e.g., weekends). **Conclusions:** Caregivers demonstrated great variability regarding implementing strategies to enhance their children’s sleep quality, suggesting that parents may be unsure of how to optimize the strategies they employ, which are most effective, or how to manage resistance from their children. Clinicians should discuss how to address these practical challenges with caregivers. Future research investigating the developmentally unique differences in determinants of sleep quality among elementary school-aged children is prudent.

## 1. Introduction

### 1.1. Overview of Sleep Health

Sleep is essential for optimal mental and physical health at all life stages [1,2]. A multi-dimensional construct, both qualitative and quantitative measures are needed to fully capture all facets of sleep [3,4,5,6]. Among children and adults, measures of duration (the amount of sleep obtained in a 24 h period), efficiency (the ease with which one falls asleep), continuity (the ability to stay asleep), timing (when one typically sleeps in a 24 h period), alertness (the ability to maintain one’s attention while awake), and satisfaction (subjective assessment of sleep quality) are all used to define sleep health. Meltzer et al. (2021) also indicate that behaviors are central for attaining and maintaining optimal sleep health [6]. These include bedtime routines, consistent bedtimes, food consumption patterns, and technology use that are primarily controlled by caregivers, rather than school-aged children themselves. Additionally, many studies of pre-pubertal pediatric sleep rely on caregivers to articulate measures of sleep constructs (e.g., continuity) as they are more involved with the sleep habits of younger children. Consequently, caregivers play a critical role in defining and understanding pediatric sleep health [6,7,8,9,10].

### 1.2. Sleep Among Elementary School-Aged Children

Sufficient or good sleep quality, defined by the CDC as “uninterrupted and refreshing sleep,” has been correlated with positive physical and mental health outcomes across the life course, such as preventing seasonal and chronic illnesses and improving attention and memory [1]. Insufficient sleep, resulting from short sleep duration (e.g., trouble falling asleep, waking up during the night) or sleep disorders, is associated with adverse physical health outcomes such as obesity and neurocognitive performance relating to memory and attention, as well as adverse emotional and behavioral health outcomes, such as depression, anxiety, and ADHD [1]. Consequently, given these developmental and potentially long-lasting health implications, sufficient sleep is especially important for children.

Sleep quality consists of multiple constructs that capture both qualitative and quantitative measures, including restoration and continuity [11] that are influenced by socio-ecological factors [12]. A commonly used measure of sleep health is duration: the American Academy of Sleep Medicine recommends that school-age children (six to 12 years) sleep between nine and 12 h each day [2], yet multiple studies indicate that insufficient sleep is prevalent among youth [13,14]. In a nationally representative sample of 112,925 U.S. children aged three to 17 years old, 34.7% experienced inadequate sleep, with the highest prevalence among children ages 6 to 12 years old (37.5%) [14]—the primary demographic examined for this paper. Another study conducted in a Southwestern U.S. school district found that nearly two-thirds of students ages 9 to 11 years (*n* = 257) did not meet the national sleep recommendations [13]. Furthermore, an additional study assessing sleep among children in the same age group identified an increasing prevalence in insufficient sleep as children age; the average percentage of insufficient sleep rose from 1.1% at age 6, to 3.9% at age 8, and 4.2% at age 10, respectively [15].

### 1.3. Factors Influencing Children’s Sleep Quality

Overall, evidence suggests that good sleep hygiene behaviors, such as regular sleep/wake times, a consistent bedtime routine, limiting screentime and certain foods close to bedtime (e.g., caffeine), are associated with sleep quality [16,17]. However, as busy lifestyles and irregular schedules become commonplace in many households, scholars are attempting to disentangle factors that lead to poor sleep quality and insufficient sleep among elementary school children. Furthermore, given the key role of caregivers in the lives of young children, researchers have sought to understand parental influences on children’s sleep quality. For example, to monitor or enforce sleep duration, families may use a range of strategies from bedroom rules (e.g., no food or screens in the room) to bedtime routines (e.g., limited activities before bed, consistent bedtimes).

Children’s sleep schedules and routines may be influenced by environmental or familial factors. For example, school schedules can also disrupt children’s sleeping patterns as they may decrease napping without a proportional increase in nighttime sleep [18]. Adjusting to a normal sleep routine can also prove difficult for children in unstructured households or those characterized by less parental control [18]. Also, parental influence on good sleep hygiene routines helps create a conducive sleep environment for children [19]. However, parental mental state and the lack of enforcing good sleeping habits can negatively affect children’s routine and sleep quality [19].

Individual health behaviors may also influence children’s sleep quality. For instance, there is a well-documented association between electronic screen use (e.g., television, video games, computers, mobile phones) and shorter sleep duration among children 6 to 12 years old [20]. While the American Academy of Pediatrics recommends that parents limit their children’s technology use, particularly one hour before bed [21], evidence suggests it is a bedtime activity for many children [21,22]. Additionally, unhealthy dietary patterns have been correlated with poor sleep quality in children [23]. Parental dietary and eating behaviors are therefore critical to sleep quality as they influence their children’s at-home eating behaviors through mechanisms such as modeling and shared meals [24].

In addition to family structure, cultural, socio-economic, demographic and environmental factors can influence sleep quality. For example, one study reported a higher prevalence of insufficient sleep among children from racial and ethnic minority groups (e.g., non-Hispanic Black (50.0%), Hispanic/Latino (40%) and low-income households (44.9%) [25]. Additionally, children who experience negative neighborhood factors (e.g., lack of safety) and family factors (e.g., inconsistent bedtime) also reported more insufficient sleep [14]. Consequently, interrogations of caregiver perceptions of sleep should include racially, ethnically and socio-economically diverse perspectives.

### 1.4. Investigating Sleep Quality in Elementary School-Aged Children

Despite extensive literature highlighting the importance of sleep, a notable gap remains in research focused on early school-aged children. Existing studies on sleep quality have largely centered on adults, with teenagers also receiving considerable attention, particularly in relation to social media and technology use [25]. Children, especially those who are elementary school-age (7–9 years), are underrepresented in sleep research in the United States and globally. As prepubescent children, their sleep patterns are highly variable due to ongoing developmental changes, and the continued development of their brains during this period complicates the identification of “normal” sleep behaviors [26]. Furthermore, parents may not consider sleep to be a primary concern for their early school-aged children, given several other pressing physical and psychological developmental challenges at this age (e.g., pubertal development, mental health) [25,27]. However, current research illustrates the importance of adequate daily sleep (9 to 12 h) for optimal health outcomes (e.g., mental, physical) in this age group [2]. Still, the effects of sleep deprivation within this age group have yet to be understood entirely, indicating that additional research is needed to support interventions on sleep habits and neurological deficits [28]. Evidence also suggests parents and caregivers overestimate how much sleep they think their children are getting each night [29]. which also points to the importance of their perspectives to account for this gap.

### 1.5. A Methodological Opportunity for Qualitative Inquiry

Prior studies have shown that many existing quantitative sleep measures fail to capture all key domains of sleep quality, underscoring the potential for qualitative inquiry in this research area [25,27]. A systematic review of objective sleep measurement instruments (e.g., polysomnography, actigraphy) found that these methods have utility in identifying sleep disorders and disturbances, but cannot fully measure all constructs of sleep health in children [27]. Thus, a qualitative approach is essential to fill these gaps. For example, research that engages parental perspectives offers insight into what factors caregivers perceive as affecting their children’s sleep quality. One study found that parents who have sleep difficulties themselves reported more sleep difficulties in their own children than parents without sleep difficulties [30]. In another study, parents perceived that good sleep quality was essential for health and aimed to create a conducive sleep environment; however, they also discussed existing barriers to fostering good sleep quality, including busy lifestyles, lack of positive parental role models, and insufficient sleep education [31]. Among children, semi-structured interviews with kids aged 5 to 6 years old found that they could describe sleep-related behaviors, emotions, and routines, such as overnight awakenings and sleep resistance [32]. Furthermore, a community-based qualitative study investigating the multidimensional influences on sleep among Latino pre-adolescents found both children and parents indicated that environmental sounds, social factors (e.g., peer conflict), screentime, and stress most negatively impacted sleep in this population [33].

### 1.6. Study Aims

This study aims to contextualize elementary school-aged children’s sleep health by qualitatively investigating caregiver perceptions of determinants of sleep quality among a racially, ethnically, and socio-economically diverse sample in the northeastern United States. This approach will address a methodological gap in the literature, enabling a deeper understanding of not only children’s sleep behaviors but also parental roles and strategies for sleep promotion. The findings from this research will provide insight into children’s sleep health by identifying factors influencing sleep quantity and quality in an age group typically underrepresented in sleep research. This study, as well as the research team behind it, represents a racially and ethnically diverse group; as such, the results may also help fill in the gaps within existing sleep research among vulnerable populations, namely Hispanic and Latino communities. This study will inform future research and interventions promoting children’s sleep health and overall wellness.

## 2. Materials and Methods

### 2.1. Participants

Participants were recruited from Project Green Space, Sleep, and Mental Health (G-SPACE), an ongoing research study examining the influence of green space on sleep and mental health among elementary school-aged children [34]. The study is conducted by the Social Epidemiology Lab at Brown University School of Public Health. Eligibility criteria for the study population are as follows: (1) legal parent, guardian, or recognized caregiver (e.g., grandparent) aged 18 years of age or older, (2) child in the first, second, or third grade (approximately 6–10 years of age), (3) ability to communicate in English or Spanish, and (4) residence in Rhode Island within selected portions of the sampling area to reflect varying levels of green space exposure. The Brown University Institutional Review Board approved all study procedures [protocol #2105002996].

### 2.2. Research Team and Reflexivity Statement

All study procedures, including recruitment, consent, interviewing, transcription, and analysis, were implemented by a team of research assistants with oversight by the principal investigator (DGT) and project coordinator (ARA). Research assistants were a mix of undergraduate students, graduate students, and full-time staff members in the Social Epidemiology Lab at Brown University. Most of the research team members self-identified as women. The team included individuals from a mix of racial, ethnic, and socioeconomic backgrounds. Three team members were bilingual in English and Spanish (APMK, MD, ARA).

At the inception of this analysis, the research team discussed the importance of reflexivity in qualitative research. To cultivate self-awareness, each individual independently completed worksheets about personal and social identity adapted from Equitable Teaching at the University of Michigan [35,36]. The personal identity worksheet includes prompts such as favorite food and birth order, while the social identity worksheet covers components such as race, (dis)ability, and sex. Afterward, the group collectively debriefed on how one’s identity may create biases or generate assumptions that influence the research process, namely, how one approaches research and interprets participant responses. For example, most team members were students and child-free. They highlighted how there may be challenges with fully understanding or interpreting responses from caregivers due to a lack of shared identity. Other research assistants negotiated how racial/ethnic concordance with participants may foster understanding of responses but could also contribute to affinity or confirmation bias. Bilingual team members discussed a similar contention regarding how their Spanish knowledge may align with, or differ from, the dialects used by study participants and therefore shape the translation process. The group also considered how racial/ethnic discordance may limit interpretation or perhaps allow for more interpretive distance, mitigating affinity bias. Additionally, because most team members were students, many did not immediately self-identify as researchers. Though prior field experience collecting quantitative data from participants—most often in their homes—helped establish rapport and cultural sensitivity, the group contended with their respective roles as “the researcher.” In particular, the team discussed how this role can create an unbalanced power dynamic that shapes participant interactions, the interview setting, and colors the analysis process. Each step of the research, analysis, interpretation, and writing processes was planned and addressed as a collective (i.e., peer debriefing) to help reduce these and any other unexamined biases.

### 2.3. Recruitment

Starting in 2021, participants were recruited through flyers distributed in schools and community spaces across Rhode Island, attendance at community events, and advertisements on radio and social media (i.e., Instagram, Facebook). Recruitment materials and all study protocols were provided in English and Spanish to ensure accessibility for all potential participants. From our sample of complete participants for the parent study as of March 2024 (*n* = 128), a cohort was randomly selected to participate longitudinally (at two more time points in different seasons from the first). Those who were excluded from the longitudinal cohort (*n* = 51) were invited to participate in qualitative interviews that took place between March and December 2024. Longitudinal participants were not invited to participate in qualitative interviews to prevent contamination within the parent study; asking caregivers about sleep strategies may potentially influence decisions during subsequent quantitative data collection. Of the 51 eligible participants contacted, 30 parents initially agreed to participate, with family time constraints noted as the primary reason for lack of participation. Our final sample of participating households (*n* = 21) includes 21 parents and 25 children. The analytic sample for this report includes all participating parents (*n* = 21). The demographic data for the qualitative cohort was taken from the Project G-SPACE parent study; this dataset includes Hispanic/Latino as a race category to capture those who otherwise did not indicate a race.

### 2.4. Study Procedures

Eligible participants were contacted via text message by the project coordinator, who informed participants that the study would involve two separate interviews: (1) a caregiver interview on their perspectives of their child(ren)’s sleep and (2) a child interview on perspectives of their own sleep. If participants demonstrated interest, the project coordinator subsequently scheduled a time for participants to complete a virtual interview with a research team member over Zoom [37]. The consent process took place electronically through REDCap [38,39] at the time of the interview, during which parents signed a consent addendum for themselves and provided permission for their children to participate. Additionally, children provided assent to participate.

Our semi-structured interview guides were adapted from a study about sleep among Latino pre-adolescents (10–12 years old) [33]. Caregivers were asked about their child’s sleep habits/patterns, household rules for sleep, and their perspective on factors that contribute to their child having a good/bad night of sleep (i.e., sleep quality) [see Additional File S1]. For example, participants were prompted: “Tell me about your child’s sleep habits/patterns,” and asked: “Think about the last time your child had a really good night of sleep. What was the day/evening before that like?” Beyond encouraging reflexivity, potential interviewer bias was managed with training. For instance, training emphasized the importance of asking all semi-structured interview questions in the same order and without embellishments. Team members conducted both the child and parent interviews in the participants’ preferred order (child or parent first) and language (English or Spanish). Pseudonyms were used to maintain the confidentiality of interviewees. The duration of interviews ranged from 15 to 20 min. The discussion was recorded using a 360-degree audio recorder (Zoom H3-VR). Participants were compensated $30 for the child and parent interviews, respectively.

Interview audio files were computer-transcribed using Philips Speech Live [40]. Interview transcripts were then manually verified, edited, and cross-checked in Microsoft Word [41] using the original audio file to ensure transcript accuracy. Spanish-language transcripts were verified by Spanish-speaking research team members. To facilitate clarity and readability, team members carefully determined that certain aspects of conversations (e.g., vocal pauses, asides) were appropriate for exclusion. Personally identifiable information was also redacted to protect the identity of the interviewees.

While this study collected both parent and child perspectives on sleep, we decided to focus the scope of this paper on the parent interviews. Although the interviews were ideally conducted separately, the parent was often present during their child’s portion(s), and thus directly and indirectly impacted their responses. At times, parents function as a second interviewer, prompting or probing their kids, and in some instances as a second interviewee, feeding them the answer. Perhaps partly due to these occurrences, the research team found similar answers and concepts present within corresponding caregiver and child(ren) sets of interviews after data collection. Additionally, conducting semi-structured interviews with school-aged children comes with inherent challenges such as contending power dynamics between interviewer and interviewee, participant stress, limited developmental appropriateness, and poor data quality [42]. Though the virtual interview format was selected to reduce participant burden—as the parent study involves seven days and multiple procedures—this format may have exacerbated these issues. Furthermore, parent perceptions have a unique utility in the context of children’s sleep health, as caregivers typically have more control over their kids’ activities and behaviors at this age and may be more likely to yield actionable insights. For these reasons, the research team decided to exclude child interviews from this analysis to reduce researcher burden, preserve analytic coherence, and focus specifically on caregiver roles in shaping children’s sleep quality.

### 2.5. Qualitative Data Analysis

Given that this study sample was drawn from an ongoing parent study (Project G-SPACE), one of the goals of this qualitative analysis was to potentially understand preliminary quantitative findings. As such, the governing research paradigm was post-positivism [43]. Template-style thematic analysis (i.e., template analysis) was completed using the method outlined by King and colleagues [44,45]. This analytical approach can be used from many philosophical positions, including work aligned with a more realist ontology [46] (e.g., operates from a post-positivist paradigm). Coding and qualitative analyses were performed using NVivo (version 15.3.3) [47]. To maintain qualitative rigor, the research team completed individual and team memos to document operational decisions, transcription and coding observations, suggestions for codebook revisions, analysis and interpretations, patterns that may be relevant for theme development, and reflexive commentary. Meeting agendas and notes functioned as an audit trail to promote dependability. The team also prioritized peer debriefing and consistent feedback from the senior author/PI to further enhance the credibility of the study findings.

To begin the analysis process, the team drafted a template that included deductive codes generated from the interview guide and inductive codes based on observations from the transcription process (i.e., data familiarization). Further codes were identified through the preliminary coding process of a sample of the interview transcripts. Codes were grouped and organized hierarchically to facilitate analysis and theme development [48]. For example, mentions of school activities, exercise, and youth programs from parents were categorized under a parent code called ‘Daytime Activities.’ Any additional changes that needed to be made to the template were identified through this preliminary coding and grouping process. The research team developed a corresponding codebook with code definitions, inclusion and exclusion criteria to assist with the coding process. The codebook also underwent multiple revisions to ensure consistency. Through this iterative coding and memoing process, group discussions, and consultation with a senior researcher, our team developed a comprehensive template and codebook that was applied to all the interview transcripts. The final template is included as an additional file [see Additional File S2].

The research team determined that a bilingual coding approach—applying the English language codebook to the Spanish language transcripts—was the best method for coding the Spanish interviews. This approach conserves conceptual equivalence, reduces translation burden, and facilitates analysis with a primarily English-fluent research team [49,50]. Spanish language quotations included in this report were one-way translated to English by bilingual research team members and cross-checked during the writing process. To preserve conceptual equivalence, translators prioritized key concepts rather than word-for-word translations.

In alignment with the post-positivist research paradigm, the team sought to ensure the codebook was applied consistently. Inter-coder reliability was assessed solely for this purpose, rather than being relied on as a central metric for qualitative rigor. Multiple coding comparison queries were run in NVivo to measure agreement among coders and check for discrepancies between coding patterns [47]. Every team member individually coded the same two transcripts until there was enough confidence in the percent agreement (>80%) to complete the full coding process [51]. The highly collaborative nature of this project facilitated shared understanding of the codebook. This reduced guessing during coding and justifies the use of percent agreement instead of Cohen’s kappa as an agreement statistic [52]. Once agreement was established, two independent coders were assigned to each remaining transcript. Though a few discrepancies emerged, all were discussed and resolved as a team. Any final calls were adjudicated by the leading author (APMK).

Transcripts were coded completely; however, they were only analyzed until code/thematic saturation was reached [53]. The research team defined saturation as the point when no new high-level concepts (i.e., parent codes) emerged. Consequently, this limited the number of potential relationships between codes that the team could identify and synthesize. Using these parent codes as a guide, multiple strategies were employed to develop themes. At the child code level, the team assessed code distribution, ran queries to assess intersections between codes, sought divergent responses, and wrote independent memos. Furthermore, the team discussed high-level patterns, considered relationships between parent codes (e.g., “Sleep Environment” and “Social Dynamics,” or “Bedtime” and “Sleep Quality”), and wrote group memos. All interpretive decisions were made collectively. We developed five themes regarding caregiver perceptions of determinants of sleep quality among their elementary school-aged children. Any subthemes or divergent concepts are captured in the reporting of these themes.

## 3. Results

### 3.1. Descriptive Statistics

Our analytic sample included 21 caregivers who were on average 39 years old (standard deviation: 8.53) and female (81%). In terms of ethnicity, approximately half of the sample identified as Hispanic/Latino (48%). Additionally, the sample self-identified race as White (33%), Black/African American (10%) and multiple (10%) (Table 1).

At the household level, the majority of households (66%) spoke English as their primary language, with the remaining third speaking Spanish (Table 2). More than half of the families had a household size of four (57%). Household incomes ranged from less than $50,000 to over $150,000 annually. Forty percent of families (*n* = 8) had a household income of less than $50,000 (Table 2).

### 3.2. Theme 1: Caregivers Report That a Busy Day for Their Children Facilitates Good Sleep Quality by Reducing Sleep Onset/Latency and Promoting Sleep Continuity

Caregivers conceptualized a “busy day” as a day packed with a schedule of different activities for their child (e.g., sports, after-school playdates). A majority of participants consistently described a relationship between a busy day and better sleep quality, regardless of the day of the week. For example, one participant mentioned, “I think the more activity that she has done during the day or the weekend, she goes right to bed, no issues.” Good sleep quality was often discussed in terms of sleep onset (i.e., how long it takes to fall asleep) and continuity (i.e., the extent to which one sleeps uninterrupted). Secondly, another participant shared, “I really have to use melatonin sometimes to get them to go to sleep, but it’s usually on days that I don’t take them outside… I try to take them out to those areas so they can run and play.” This underscores the importance not only of using outdoor green space, but also of children’s sleep quality, as shown through active stimulation, such as play and activity.

Most caregivers explained that their children had better sleep quality with higher levels of physical activity throughout the day. For example, one Spanish participant shared, “*Tuvo demasiada actividad física, que estaba súper cansado. Y él en una noche sí que durmió bien. Fue porque lo llevamos a nadar y dice que estaba cansado*.”/“He had too much physical activity, so he was super tired. And that night he actually slept well. It was because we took him swimming, and he says he was tired.” Many caregivers discussed how regular sports activities, such as games and practice, can drain the child physically, allowing for better sleep quality. For example, a participant shared that when their child has soccer games, they “[feel] tired and go to sleep a little bit earlier.” However, physical activity did not always refer to sports or other vigorous activities. For example, one caregiver stated, “Like I remember one time we went to the zoo and it’s a lot of walking. It was like, in [the] hot sun and both of them slept. Both of them slept really well.”

Furthermore, caregivers reported that not only physical, but also mental tiredness led to reduced sleep onset/latency and promoted sleep continuity. For example: “I think if she’s really like had a day filled with activities and she’s like active and like moving her body a lot and her mind is just so tired she knocks out, and she’ll sleep through the night.” Emphasizing mental tiredness stemming from cognitive engagement, a caregiver stated, “There’s so much going on daily for them… with your brain, with what you’re learning at school… it’s all draining… whether your body is physically tired or your brain is mentally tired.” This illustrates the role of mental activity during a busy day and its impact on sleep quality.

### 3.3. Theme 2: Caregivers Employ a Variety of Screentime Rules as a Strategy for Improving Their Children’s Sleep Quality

Participants were asked about their expectations regarding their children’s use of screens (e.g., phones, tablets, TV, video games) before bedtime. These rules were highly variable throughout the participants, potentially reflecting differences in preferences or underlying constraints such as parental uncertainty or household negotiation. Children use screentime for a variety of different purposes. One participant commented that “there’s a blurred line between when screentime is like for recreation and when it’s like for school…” This describes parental ambiguity around the purpose of screentime. This distinction is important for a parent’s decision process regarding the allowance of screentime before bed. Moreover, many family routines were found to consist of screentime before bed. As one participant shares: “We’ll sometimes watch a movie together or something, read and then bed, brush their teeth, all that stuff in between, but very routine and go to bed. They do watch TV before bed. They do have TVs in the bedroom…”

Most caregivers implemented rules for screentime, but the extent of these rules varied greatly. Some caregivers described enabling parental controls on their child’s phone or other electronics to limit the duration of screentime. One participant shared, “All of the electronics are controlled remotely in the sense that you know, they shut down at a certain time, and there’s just no access to them after that.” Another finding was the struggle to control screentime:

“My daughter does have an addiction to electronics and gaming and friends. So it’s kind of tough trying to, you know, pull and get that detachment away from the electronics. Trying to give her a cut-off time so that way she can wind down before bedtime. Because I don’t want her thinking about it, I don’t want her having those thoughts in her mind, the game or of the electronics.”

Other participants also described their child’s addiction to their screens, making it difficult to create boundaries and rules. Like this quote, a participant stated that “…we try to limit screentime before bedtime because I know that it can like, make her brain like, go crazy.” This demonstrates the perceived negative mental effects of screentime on children.

Caregivers also explained various strategies to control the use of electronics, specifically in their child’s sleep environment, ranging from complete elimination to limited screentime. For example, some participants explained electronic devices were not in their child’s bedroom at all: “So no screens in their bedroom…” Or as another participant outlined, “…there’s no electronics in the room, period. There’s no TV, there’s no tablet that sits there, nothing. Everything has to be dark, closed door, quiet, relaxing.” On the contrary, some caregivers shared that their children did have electronics in their bedrooms, but with rules about usage: “*Nuestra hija tiene un iPad y un TV en su habitación, pero los apagamos a la hora de acostarse*.”/“Our child has an iPad and a television in her bedroom, but we turn them off when it’s bedtime.” Taken together, these parental accounts elucidate the ongoing negotiation between caregiver authority and children’s increasing autonomy, particularly in technology use.

### 3.4. Theme 3: Caregivers Employ a Variety of Food Rules as a Strategy for Improving Their Children’s Sleep Quality

Caregivers were asked about their expectations regarding their children’s food intake before bedtime. In response, participants often described where, when, and what their child was allowed to eat. Often, the goals of these food rules were to maintain a clean sleep environment and promote good sleep quality. Caregiver descriptions suggest that food rules work to structure routine as well as manage children’s behavior. Overall, participants demonstrated diversity in food rules around bedtime. While most caregivers enforced some kind of restriction, particularly surrounding eating in bed and consumption of sugary foods, their level of strictness varied. This variability emphasizes that caregivers do not uniformly adhere to sleep hygiene recommendations but navigate these parenting styles considering their household preferences and constraints.

Several participants emphasized rules prohibiting eating in the bed or bedroom altogether. One participant stated, “Yeah, there’s no eating in the room, period.” This underscores a firm boundary around food in the sleep environment. Another participant similarly noted, “*No, no se permite comer en la cama*”/“No, we don’t allow eating in bed,” going on to explain that although children may eat in their room, it must be at a desk or table and never on the bed. This represents a level of variability in food rules. These rules were often motivated by concerns about cleanliness and/or bedtime routines to keep the bed as a space purely for sleep. For example, one caregiver shared that food was not allowed in bedrooms due to issues with pests.

Caregiver rules about the timing of food intake before bedtime also varied. Some participants reported that children were not allowed to eat after dinner, except for water. For example, *“Normalmente no más… las dejamos que tomen agua, si es que ya van a la cama, solo agua. No merienda, no jugos, no nada.”*/“Normally no more…we let them drink water, if they are already going to bed, only water. No snacks, no juice, no nothing.” Other caregivers allowed bedtime snacks depending on how late dinner was. One participant explained, “If it’s a later night… then no, you’re not having a snack.” This variation suggests that food timing rules are negotiated depending on the schedule of the day, highlighting the flexible nature of caregiving practices.

The type of food allowed before bed differed between families. A common theme among participants was sugar intake before bed. One participant commented, “I think being on that, being on that sugar high, I guess like late at night or whatever, and then not falling asleep at the right time and still having to get up early makes for like not feeling restful.” Several caregivers reported limiting sugar intake in the evening to avoid sleep disruption. One participant stated, “…we try to eat less sugar and more fruits, more protein.” Another noted, “…as far as other rules, you know, we don’t do sugar too close to bedtime,” explaining that sugar immediately before bed could interfere with their child’s sleep quality. In contrast, some participants shared that having a sugary snack positively changed their children’s mental state before bedtime: “…last night it was all lovey dovey ‘cause she had a milkshake at McDonald’s last night. She was all lovey dovey when she went to bed.” Further, some caregivers substituted sweets with healthier alternatives, such as “eating a bigger snack at bedtime that’s a bit more protein-based,” particularly if they believed the child was truly hungry and not feigning hunger to stall bedtime. Another method to avoid sugary desserts was eating a late dinner. A caregiver described, “So we try if we have a late dinner, like they’re, they need dessert every night, but the dessert will look like an apple if it’s a later dinner. If we eat earlier, sometimes they’ll have that little scoop of ice cream or a cookie or something like that.” Ensuring a late dinner was seen to promote healthier dessert alternatives in this family. Overall, this represents the varied perceived effects of food before bedtime.

### 3.5. Theme 4: Caregivers Observe That Sibling Dynamics Disrupt Their Children’s Sleep and Wake Times

Caregivers frequently described sibling relationships as a significant factor influencing children’s sleep/wake timing and ultimately, sleep quality. These influences were both direct (e.g., shared bedrooms, co-sleeping) and indirect (e.g., social dynamics). Most participants explained that sibling dynamics disrupt sleep routines.

Several caregivers noted that one child’s mood or behavior could delay sleep onset for their siblings due to interactions such as conflict or play. For example, one participant explained their children’s shared sleep environment: “And she shares a room with her sister, so sometimes they play around and talk and don’t go to bed right away. I think the problem is that she shares her room with her sister and they love to argue and fight and it’s constant.” The participant goes on to explain that this leads to delayed sleep onset. Another caregiver mentioned, “…if my youngest is like, rambunctious, they all stay up. But it really all depends on her if they’re calm. It’s easier for them to go to sleep.” These quotes explain that evening sibling interaction and energy levels are key components of delayed sleep onset. Another mechanism in which siblings impact sleep quality is the difficult balance for caregivers to accommodate different sleep preferences and best promote sleep quality. One caregiver stated that adjusting to their children is “…a challenge on my part and something I’m working on. But just what parents can do to actually help kids ‘cause they’re all so different no matter what you do.” This highlights a struggle in discovering the best methods to help their children sleep with various needs.

Across the shared experiences among participants, there was a balance between siblings influencing delayed sleep onset at bedtime and impacting morning wake-up times. One participant states that their child wakes up “…because she heard my older daughter and she wakes up earlier, and that’s why she wakes up.” This shows that sibling presence in or near the sleep environment can cause disruptions to sleep continuity. On the other hand, some caregivers implement household rules to ensure that siblings do not wake each other up. A participant describes that a “big rule is not to be waking up her little brother, and he’s not supposed to be waking her up.” This highlights how one sibling’s behavior can influence and disrupt or maintain sleep routines across the whole family.

### 3.6. Theme 5: Caregivers Tend to Prioritize Regular Sleep/Wake Times for Their Children During School, but Many Allow More Flexibility on Weekends and in the Summer

Caregivers were asked about their children’s sleep habits and patterns, in addition to any rules about sleep/wake times for their children. Most participants described that they enforced consistent bed and wake times for their children. If not a specific time, caregivers might refer to a time window or range: “Yeah, there’s not like a set like ‘you need to be asleep by this time.’ But I mean, I’d say it’s like a window of time.” In many cases, caregivers described this enforcement of sleep/wake time as a product of their children’s school schedule. This especially arose when discussing wake times, as one parent explained, “*…entre semana, como ellos tienen que ir a la escuela, [despiertan] como [a las] 6:00, 6:30, por ahí*.”/“…during the week, since they have to go to school, they wake up at like 6:00, 6:30, somewhere in there.” Although some caregivers stated that the regularity of their children’s sleep and wake times during the school week also extended to the weekend. One parent claimed, “It’s relatively the same. So like, you know, falling asleep usually between 8:00 and 9:00 pretty much all seven days of the week…We try to keep it pretty consistent.”

Other participants explained that their children’s sleep/wake times varied on the weekends compared to weekdays. Some reasons for these differences included changes in schedules/activities or less enforcement of a specific bedtime. For example, one participant outlined how other aspects of the family’s routine can influence a child’s bedtime routine: “And sometimes it’s kind of dependent upon like when I’m getting home from work or when they were able to get in the shower, that sort of thing.” In most cases, caregivers described that they allowed their children to go to bed later on non-school nights (e.g., Friday, Saturday). As this participant elaborated: “*…el viernes…se duerme un poquito más noche como a las 11, porque a veces dormimos más noche como 11 o 12…*”/on Fridays…he sleeps a little bit later like at 11, because sometimes we sleep later like 11 or 12…” Or as a different parent explained, “Weekend night is just like everything’s a little bit longer…So it’s just like we’ll allow her to do the same routine I suppose, but like allow more time for each.” Several participants explained that a later bedtime could lead to their children sleeping in the following morning, but many also claimed their children woke up at the same time on the weekends: “I have to say a bad night of sleep to me for her is going to bed late because she wakes up at the same time, if not earlier.” Other caregivers also found that their children woke up earlier on weekends. For instance, a parent said, “If she has an early sporting event that I have to wake her up for, she’s fine. Like, even if it’s earlier than school, wake up…” explaining that her daughter wakes up an hour and a half earlier on Saturdays compared to school days.

Some participants noted that their children’s sleep/wake habits can be subject to seasonal changes in the summer. One parent articulated changes in their child’s bedtime, “Yeah, I’m pretty consistent between 8:00 and 8:30 on school days and weeks. No school, usually around 8:30 to 9:00. But I’d say it’s more seasonal. So summer when there’s more things going on, you know, a little bit later than that, but I’d say right now in the winter months, it’s not too far off from what school is.” Similarly, a different parent spoke of their flexibility around wake time despite a consistent summer schedule, “…once we start summer camp and things of that nature that he’s going to have to get up like he was going to school because he’s still getting dropped off, then we go back to the bedtime routine. But we’re lenient during the summer because it’s camp.” Another caregiver elaborated on this idea, highlighting that not only one’s schedule, but also one’s response to seasonal changes may influence bedtime: “…in the summer it’s a little bit harder to get her down because the sun is still up for such a longer time after that 7:30 time. So making sure that her bed or her room is as dark as possible, that’s what I try to aim for.”

Some caregivers emphasized the importance of a specific number of hours of sleep per night instead of specific sleep/wake times to enhance their children’s sleep quality. One caregiver explained how their son makes up their requisite hours of sleep after a late night:

*…a veces…nosotros tenemos alguna fiestecita y a veces nos dormimos a las 2:00 de la mañana o 3 de la mañana, él [no] se duerme hasta esa hora. Pero también al otro día se levanta tarde...como las 1 p.m., que él necesita. Él completa su[s] hora[s] de sueño en los fines de semana*./…sometimes…we’ll have a little party and sometimes we will go to sleep at 2:00 in the morning or 3:00 in the morning, he does not fall asleep until that time. But also, the other day we will wake up late…like at 1 p.m., because he needs it. He completes his hours of sleep on the weekends.

Similarly, another participant highlighted the importance of setting a limit to how late their children stay up to promote adequate sleep time: “*A veces…ceden a dormirse un poco más tarde porque es fin de semana, pero no…tan tarde, no. Porque igual, pues ellas tienen que dormir su tiempo completo…*”/“Sometimes…they give in to sleep a little later because it’s the weekend, but no…so late, no. Because [it’s the] same, well they need to sleep their complete time…” Some parents explained that going to bed late had consequences for their children who tend not to sleep in: “It is like I said, there is not many times where, you know, bad night sleep [is] like ‘oh, she’s up in the middle of the night’ and all of that. It more so to me is like the duration.” In other words, kids who cannot “make up” their lost sleep time can often be tired or irritable the next day.

## 4. Discussion

Our findings highlight that caregivers believe that children’s daily routines and social dynamics play a central role in sleep quality. We found that parents reported that both physical and mental tiredness promote sleep onset and improve sleep continuity. Caregivers often described “busy days” that include sports and after-school activities. Caregivers also reported a variety of screentime rules in hopes of promoting sleep quality. This can be seen through screentime limits and parental app controls. Also, food rules were often reported as a means of enhancing sleep quality (e.g., limiting sugar before bed, not eating in the bedroom). Sibling dynamics were another important factor in sleep quality, as they were seen to disrupt bedtime routines and wake-up times. Finally, caregivers reported efforts to maintain regular sleep and wake times for their children throughout most of the year; however, they extended more flexibility on weekends and in summer months. This flexibility sometimes reflected prioritization of total hours of sleep (i.e., “catching up”) rather than consistent sleep–wake schedules.

### 4.1. Conceptualizing ‘Activity’ as Both Physical and Mental

A common theme found among participants was that a busy day for their children influenced sleep quality positively, meaning faster sleep onset and greater sleep continuity. This is consistent with previous research on physical activity and sleep among children and older adults. Specifically, individuals engaging in at least 30 min of exercise a day slept on average 15 min longer than those who did not [54]. Additionally, physical activity significantly contributes to maintaining balance in the body’s sleep–wake cycle, which affects sleep quality, making it an important factor to study among children. 

One systematic review emphasizes the benefits of physical activity and its effects on sleep disorders. It was shown that active people had higher levels of positive tranquility, which helps regulate body temperature, a necessity for faster sleep onset [55]. However, our findings suggest that not only physical activity, but also mental activity can contribute to good sleep quality. This is consistent with mental stimulation research in older adult populations. Studies show that brain stimulation, such as hippocampal activity and transcranial electrical stimulation, has been associated with enhanced sleep quality among adults [56,57]. In our study, the concept of “mental tiredness” in addition to physical activity refers to the exhaustion from a full day of stimulation, whether it be cognitive exertion with schoolwork or emotional processing throughout the day. For school-age children, there is limited research regarding the link between mental stimulation and sleep quality, making this a novel construct specific to caregiver perception that warrants further exploration. This underscores an important contribution of this study to the understanding of pediatric sleep.

### 4.2. Improving Implementation of Caregiver Rules to Enhance Sleep Quality

Our results found that caregivers utilize a variety of rules for screentime, food, and sleep/wake times to enhance children’s sleep quality. In fact, most caregivers we interviewed implement rules to support good sleep hygiene habits, suggesting that they are informed about best practices for sleep hygiene. However, our analysis reveals that some parents experience resistance from their children when implementing these practices. This finding points to a need to move beyond efficacy studies to explore the effectiveness of sleep interventions in real-life situations [58]. Furthermore, there is a need to equip caregivers with evidence-based strategies to handle bedtime resistance. This will provide parents with practical, evidence-based recommendations to manage their children’s sleep quality.

Screentime before bedtime has been shown to have a variety of deteriorating effects, specifically poor sleep quality [59]. For example, a cross-sectional school-based study with adolescents (aged 11–16) found an association between poor sleep quality and high screentime in over 52% of the participants [60]. These results draw attention to the importance of reducing screentime usage before bedtime, specifically among school-aged children. Moreover, our study demonstrated that parents were attempting to limit screentime usage [60]. However, parents may be unsure how to implement or optimize these strategies, which suggests there is a gap between knowledge and practice. Some families within our sample discuss their children having an “addiction to electronics” which creates resistance from children. This barrier reflects the practical constraints within family dynamics and the struggle to work through children’s resistance to strategies to enhance sleep quality [58]. A systematic review and meta-synthesis examining the parental perception of children’s screentime underscored parental awareness of the potential harms and need to limit screentime, with one reporting that screentime is a pacifier for children [61]. One strategy parents used was to monitor screentime with parental controls on devices, allowing them to limit their children’s screentime usage before bedtime. However, there is limited research among our age group on the feasibility and effectiveness of parents limiting screentime before bedtime [62]. A randomized clinical trial assessed the implementation of a screentime intervention with toddler-parent dyads two hours before bedtime [62]. Their results demonstrated high feasibility and efficacy within intervention adherence and positive family experience. This highlights the importance of implementing screentime interventions, especially starting early in children’s developmental period [62,63,64].

Furthermore, our findings demonstrate that caregivers employed a variety of food rules at bedtime as a strategy to improve their children’s sleep quality. Research has revealed the influence of parents on children’s eating behavior, including children’s access and availability to food [65,66,67]. One of the food rules implemented by the caregivers in our sample was limiting sugar intake before bedtime. While this is a highly studied domain, research is limited regarding the potential impact of limiting children’s sugar intake before bedtime in elementary school-aged children, specifically. However, it is important to highlight that there was also variability among the results when discussing food rules at bedtime. This could be due to different participants’ demographics and cultures, a lack of knowledge regarding the impact of food consumption before bedtime, or different dietary priorities. Some families were aware that limiting sugar consumption before bedtime was a beneficial strategy, while others demonstrated food consumption was simply based on the activity the child had per day, emphasizing the constraints in enforcing food rules. This large distinction is of great importance for intervention design when targeting children and food consumption at bedtime.

A bedtime routine has consistently been shown to support positive sleep outcomes in early childhood, such as earlier bedtime, lower sleep onset latency, lower wake after sleep onset (WASO), longer sleep duration, and improved sleep quality [17]. Good sleep habits include not only the contents of the routine, but also the regular schedule of sleep and wake time. A systematic review analyzing the effectiveness of children’s sleep hygiene techniques highlighted that a consistent timetable of sleep and wake times helps improve children’s sleep patterns [58]. Caregivers may have limited exposure to information about the significance of bedtime and consistent routines for sleep, since American pediatricians typically receive minimal training in advising parents on strategies to improve their children’s sleep [58]. During school days, caregivers enforce regular bed and wake times tied to school schedules; however, they allow flexibility during the weekends and in the summer due to a variety of factors. For example, participants discussed how in the summer, the sun is out for longer, often delaying bedtime due to the abundance of daytime activities. This is also consistent with national trends for sleep norms among children and adolescents, reflecting “social jetlag” where weekends might be used to compensate for shorter sleep duration during the week or social commitments (e.g., family gatherings) that may be more difficult on weekdays [68]. Interestingly, Ferguson & Moreno (2023) have also found that the more relaxed bedtimes for children over the summer resulted in reduced parent–child conflict, albeit later bedtimes [69]. Early-bed/early-rise patterns have been associated with more favorable health outcomes, such as lower obesity rates, while the late-bed/late-rise pattern was associated with more behavioral problems [70]. This can be related to parents in the study allowing their children to revert to a late-bed/late-rise routine on the weekends to catch up on sleep, while the early-bed/early-rise pattern is more pressing for school nights [70]. Outside of inconsistent sleep and wake times, school-age children are also not getting enough total sleep, ranging from 25 to 50% of children getting insufficient sleep in 2020 to 2021 [71]. A study conducted at Brown University highlighted that the vast majority of parents falsely believed their child was meeting the national sleep recommendation, underscoring a gap in understanding [29].

### 4.3. Investigating Familial Dynamics Beyond the Parent–Child Relationship

The findings of this research emphasize the importance of investigating sleep quality beyond the individual child with a lens toward family and/or household dynamics. The family unit is a crucial social factor that influences sleep during childhood [72,73]. However, the majority of studies on this topic assess these factors in early childhood and adolescence and, therefore, may not be directly comparable to our findings. There is also limited intervention research on the role of the family in sleep, specifically [74]. This also highlights an opportunity to conduct further research and implement family-based interventions to promote sleep hygiene and quality with an emphasis on early school-aged children.

There is an abundance of evidence that the parent–child relationship influences children’s sleep quality. In early childhood (1–3 years old), high parental presence at sleep onset (i.e., bedtime), high parental involvement in child sleep (e.g., rocking, settling), and feeding to sleep have been associated with poorer sleep quality [75]. Similarly, more negative parenting style, parent presence at bedtime, greater family problems, and family stress are documented risk factors of child behavioral sleep problems in children aged 1–10 years old [76]. Among adolescents, the parent–child relationship is also an important factor [77]. For example, optimal parenting behaviors (e.g., autonomy-granting, structured) have been shown to be moderately associated with improved sleep outcomes, including less daytime sleepiness, earlier bedtime, shorter sleep latency, longer sleep duration, and fewer sleep disturbances [77]. Additional work should explore whether there are any developmentally specific factors related to the parent–child relationship in early school-aged children worth intervening on to promote good sleep quality in this population.

Most studies on the effect of family structure/dynamics on children’s sleep quality have focused on the role of parents; however, our analysis reveals siblings may also be significant contributors. There are limited studies investigating the role of siblings in sleep quality [78]. Existing evidence is mixed regarding whether having siblings is associated with poorer sleep outcomes. For example, one study found that among children ages 4–8 years old, the parental challenge of getting their younger child to bed on time when a sibling has a later bedtime was associated with shorter sleep duration [79]. Among studies with adolescents (11–18 years old), some indicate that having siblings may lead to later bedtimes [80]. Longitudinal evidence does not support an effect of having siblings on adolescent sleep duration, bedtime, or wake time; however, it does show that children with higher levels of sibling conflict tend to experience shorter sleep duration and more sleep issues over time [78,80]. In our sample, shared sleep/wake time was found to be most disruptive to the caregivers with regard to the sibling relationships. In conjunction with the literature surrounding shared rooms or co-sleeping with siblings, it was reported to hinder sleep quality and length, especially staying and/or falling asleep [72]. Siblings with substantial age gaps may also influence enforcing consistent bedtimes, as older kids might be allowed to go to bed later compared to their younger siblings [81,82]. More research is needed to clarify the role of siblings on sleep quality among elementary school-aged children.

### 4.4. Implications for Future Research

Findings from our analysis emphasize gaps in the body of literature specifically investigating determinants of sleep quality among elementary school-aged children. While current evidence-based recommendations for enhancing sleep quality for other age groups may reasonably apply to school-aged children, it is important to discern whether there are any significant developmental differences for which parents and scientific interventions should account. In particular, there are opportunities to explore mental activity and the role of siblings as determinants of sleep quality. Given the limited transferability of interview results, future research should incorporate qualitative methods to continue to obtain caregiver perspectives. Additionally, qualitative methods can be used to elucidate the unique lens of children themselves to enhance the depth of our knowledge on sleep quality in this age group.

Furthermore, the inclusion of caregiver and child perspectives has potential implications for practical recommendations and even clinical interventions for children’s sleep health. As referenced, organizations such as the Centers for Disease Control and the American Academy of Sleep Medicine already offer guidelines and guidance for children’s sleep, but, as the findings from this study suggest, there are differing strategies being employed by caregivers to try to achieve healthy sleep. Our team suggests that incorporating regular, structured conversations around these strategies, especially with their child’s clinician, could help fill in the gaps between sleep recommendations and realistic implementation of healthy habits.

### 4.5. Strengths and Limitations

This study had multiple strengths, particularly with respect to the methods and analysis. The interview format replicates an everyday conversation and, therefore, is well-suited to elicit personal thoughts and beliefs [83]. Our study addresses a population gap by interviewing individuals who are part of an understudied group: caregivers of school-age children. Both English- and Spanish-language interview guides were used, enabling recruitment of a culturally diverse sample that reflects the population within Rhode Island. In addition, our diverse and bilingual research team fostered rapport with participants, promoting the elicitation of valid responses. Our team demonstrated a high level of inter-coder reliability throughout the analysis process. Quality was ensured by working independently while seeking to achieve agreement through discussing revisions and observations together. We also employed an independent scrutiny approach with a senior researcher to cross-check all major steps of the analysis process [84]. All operational, coding, and analytical decisions, in addition to observations, were maintained with an audit trail consisting of meeting notes and memos. Our team also consulted the consolidated criteria for reporting qualitative research (COREQ) checklist to guide reporting of this study, checking off 27 out of 32 items (84%) [85]. The five items not checked off were: 18 (repeat interviews), 20 (field notes), 23 (transcripts returned), and 28 (participant checking).

The qualitative sample was also reflective of the parent study at the time of data collection, with similar percentages of Latinos (47% sample, 44% parent study), and income levels (40% with incomes less than $50,000 in the sample, 37% in the parent study) (Table 2). However, since there was an intentional effort to recruit a racially diverse sample, the racial composition differed between the sample (33% White, 9.5% Black) and the parent study (47% White, 4.9% Black).

Notwithstanding, this study was limited in the data collection and analysis phases. Interviews favor depth over breadth, limiting our sample size and the transferability of our findings. Additionally, there are selection bias concerns, given that participants were recruited from a larger parent study; this includes non-response bias as well as selection bias from the original sampling of the larger parent study. Furthermore, we must acknowledge that the interview setting itself may make it challenging for participants to be fully transparent [83]. Although our research team encompassed diverse academic stages, we incorporated an applied learning approach where peer mentorship and training were administered throughout the analysis process, limiting the impact of differences in interviewer style and probing. The impact of additional challenges encountered during interviews, such as interruptions (e.g., children interjecting), could have also been reduced with more training to help interviewers bring speakers back to focus. Furthermore, our team experienced some challenges balancing asking open-ended questions while also obtaining data that was interpretable. For example, we asked: “What would you say affects your child’s sleep most?” Interviewers probed participants differently based on their understanding of that question. Heterogeneous interpretations of study concepts, vocabulary, and operationalizations of interview questions may have led to mixed results. Given the centrality of family processes for engendering healthy childhood habits [86], we could have spent more time probing other aspects of family life. For example, measures of family disorganization, such as limited structure and enforcement for routines such as mealtimes or parental stress, have been shown to adversely impact child sleep [87,88]. Overall family functioning in various aspects of children’s lives, including caregiver support, influences children’s sleep and other health habits.

## 5. Conclusions

This study investigated an under-researched topic: caregiver perspectives on determinants of sleep quality among their elementary school-aged children. Our findings show that parents perceive busier days—consisting of physical and mental activity—promote better sleep quality for their children. Additionally, caregivers revealed that they use a variety of strategies to help enhance their children’s sleep quality, including regulating screentime and food consumption before bed and prioritizing regular sleep and wake times. However, parents allowed their children more flexibility with regard to sleep and wake times on weekends and in the summer months; thus, there were notable inconsistencies in terms of how caregivers employed strategies to enhance sleep quality. This variability suggests parents may need further support on how to optimize sleep strategies, including how to manage resistance from their children and navigate sibling dynamics. Importantly, the perspectives that emerged from this study emphasize the depth of information regarding sleep quality in elementary school-aged children that can be discerned from qualitative data compared to quantitative measures. Future research should adopt a qualitative approach to further investigate the determinants of sleep quality among elementary school-aged children, potentially discerning factors such as developmental differences that could further shape intervention design and implementation.

## Figures and Tables

**Table 1:** Demographic Characteristics of Caregivers in Project G-SPACE ^1^ Qualitative Sample.

Demographic Characteristics	Caregivers (*n* = 21)
**Gender Identity**	
Female, *n* (%)	17 (80.95)
Male, *n* (%)	4 (19.05)
**Hispanic/Latino**	
Hispanic/Latino, *n* (%)	10 (47.62)
Not Hispanic/Latino, *n* (%)	11 (52.38)
Race/Ethnicity **	
Black/African American, *n* (%)	2 (9.52)
Hispanic/Latino, *n* (%)	9 (42.86)
White, *n* (%)	7 (33.33)
Other, *n* (%)	1 (4.76)
Multiple, *n* (%)	2 (9.52)
Age *, mean (SD)	39.55 (8.53)

^1^ Project Green Space, Sleep, and Mental Health (G-SPACE) is an ongoing research study examining the influence of green space on sleep and mental health among elementary school-aged children. * Calculated based on *n* = 20 due to one missing response. ** Percentages for race/ethnicity sum to over 100% as each question was asked separately.

**Table 2:** Household Information of Project G-SPACE ^1^ Qualitative Sample.

Household Characteristics	Households (*n* = 21) (*n*, %)
Income *	
Less than $50,000	8 (40)
$50,000-$99,999	2 (10)
$100,000-$149,000	5 (25)
$150,000 or More	5 (25)
**Size**	
3	3 (14.29)
4	12 (57.14)
5	5 (23.81)
6	1 (4.76)
**Primary Language**	
English	14 (66.66)
Spanish	7 (33.33)

^1^ Project Green Space, Sleep, and Mental Health (G-SPACE) is an ongoing research study examining the influence of green space on sleep and mental health among elementary school-aged children. * Calculated based on *n* = 20 due to one missing response.

## Data Availability

The data presented in this study are available on request from the corresponding author, as the data collection for the parent study is ongoing.

## Supplementary Materials

The following supporting information can be downloaded at: Additional File S1: Parent interview questions can be downloaded at: https://docs.google.com/document/d/13wG5I49XbvrF3sFCKskaM_h5m3MB-Mg9MaoG_DlBVxA/edit?usp=sharing; Additional File S2: Final template can be downloaded at: https://docs.google.com/document/d/1wH841wfFG_VHo6ggB2ppB2h_3WXZWl377ZBgo8h063M/edit?usp=sharing.
